# LHPP suppresses bladder cancer cell proliferation and growth via inactivating AKT/p65 signaling pathway

**DOI:** 10.1042/BSR20182270

**Published:** 2019-07-30

**Authors:** Yansheng Li, Xin Zhang, Xiaoguang Zhou, Xiaodong Zhang

**Affiliations:** 1 Institute of Urology, Capital Medical University, Beijing, China; 2 Department of Urology, Capital Medical University Beijing Chaoyang Hospital, Beijing, China

**Keywords:** AKT, bladder cancer, glycolysis, LHPP, p65

## Abstract

Bladder cancer (BC) is one of the commonest malignancies in the urinary system. Recent evidences have shown that Phospholysine phosphohistidine inorganic pyrophosphate phosphatase (LHPP) serves as a tumor suppressor in hepatocellular carcinoma and cervical cancer. However, little is known about its function in BC. Here, we aimed to investigate the role of LHPP in BC. We found that LHPP was down-regulated in BC tissues and cells. Knockdown of LHPP promoted the proliferation and growth of BC cells T24 and 5637. Inverse results were observed in SW780 and BIU87 cells with ectopic LHPP expression. LHPP also repressed the glycolysis of BC cells. At the molecular level, LHPP silencing led to enhanced phosphorylation of both AKT and p65, as well as up-regulation of their downstream targets Bcl-2 and Cyclin D1. Inhibition of AKT by MK2206 blunted the increased phosphorylation of p65 caused by LHPP knockdown, suggesting that LHPP silencing activated p65 through AKT. Importantly, p65 inhibitor (caffeic acid phenethyl ester) exhibited larger suppressive effect on the proliferation of LHPP knockdown BC cells as compared with Ctrl cell. Our study demonstrates that LHPP suppresses BC cell growth via inactivating AKT/p65 signaling pathway.

## Introduction

Bladder cancer (BC), which generally originates from the epithelium, represents the fourth most frequent malignancy in men and the ninth commonest cancer in women [1]. The incidence of BC continues to increase in the past decades that 76960 new cases were predicted to occur in the United States in 2016 [2]. Urothelial carcinoma belongs to the most common subtype of BC [3]. The routine therapy for BC patients is endoscopic resection, whereas the outcome is unsatisfactory [4]. Therefore, revealing novel signaling pathways or the molecular mechanisms responsible for BC development is indispensable for the anticancer therapy.

Dysregulation of kinases or phosphatases, such as PI3K, AKT, PTEN or DUSPs, is widely observed in cancers. Phospholysine phosphohistidine inorganic pyrophosphate phosphatase (LHPP) is conserved from worm to human with a 28% identity [5]. It is originally discovered in swine brain tissue [6,7]. Recently, LHPP has been identified as a tumor suppressor gene in hepatocellular carcinoma (HCC) by targeting PI3K/AKT signaling pathway. It is lowly expressed in HCC specimens and its low expression correlates with the poor survival of HCC patients [5]. In another study, LHPP is also down-regulated in cervical cancer and silencing of LHPP promotes the progression of cervical cancer [8]. However, the role of LHPP in other cancer types, such as BC, is largely unknown.

In the present study, we aimed to investigate the involvement of LHPP in BC. The TCGA database showed that LHPP was down-regulated in BC tissues. We further demonstrated the reduced protein and mRNA expression of LHPP in BC tissues. Functional studies identified that knockdown of LHPP enhanced the viability and colony formation of BC cells, and inverse results were observed in LHPP overexpressed BC cells. Glycolysis was inhibited by LHPP. We also revealed p65 signaling as the downstream effectors of LHPP, which is depending on AKT activity. Our findings indicate that LHPP serves as a tumor suppressor in BC.

## Materials and methods

### Patients and specimens

All the BC and normal tissues (located >3cm from the tumor) were collected from the patients who did not receive systemic treatment in the Beijing Chaoyang Hospital between May 2013 and August 2017. Each patient provided a written informed consent for the sample collection and molecular analysis. The research has been carried out in accordance with the World Medical Association Declaration of Helsinki. The study protocols were approved by the Ethics Committee of Beijing Chaoyang Hospital, the Capital Medical University. The tumor and normal tissues were subjected to Western blot and qRT-PCR analysis of LHPP expression. GAPDH serves as internal control.

### LHPP expression analysis from TCGA database

Transcriptional level of LHPP in BC tissues was analyzed from The Cancer Genome Atlas (http://cancergenome.nih.gov) database. A total of 407 BC specimens and 19 normal tissues were available for the present study.

### Antibodies and other reagents

Antibody against LHPP was purchased from Proteintech. Antibodies against p-AKT (Ser473), AKT, p-p65 (Ser536) and p65 were from Cell Signaling Technology. Antibody against β-actin and all the secondary antibodies were obtained from Santa Cruz. AKT inhibitor MK2206 and p65 inhibitor caffeic acid phenethyl ester (CAPE) were purchased from Selleck.

### Immunohistochemistry staining of LHPP

Formalin-fixed and paraffin-embedded (4 μm), BC tissues and the adjacent muscle and mucosa tissues were deparaffinized in xylene and hydrated in graded alcohol. Citrate buffer (pH = 6) were used for antigen retrieval and 3% hydrogen peroxide was used for blocking endogenous peroxidase. After incubated with 10% serum for 60 min, the slides were incubated with LHPP primary antibody at 4°C overnight and with secondary antibody at room temperature for 30 min. DAB served as a chromogen.

### Cell culture

Human BC cell lines (T24, SW780, 5637, J82 and BIU87) and normal uroepithelium cell line SV-HUC-1 were purchased from the Cell Bank of the Chinese Academy of Sciences (Shanghai, China) and American Type Culture Collection (Manassas, VA, U.S.A.). All the cells were grown in RPMI 1640 or Dulbecco modified Eagle’s medium (Gibco) medium, which was supplemented with 10–15% fetal bovine serum (Gibco) and 1% penicillin/streptomycin solution (Corning). The cell cultures were maintained in a 37°C incubator with 5% CO_2_ atmosphere.

### Lentivirus mediated overexpression and knockdown

For over-expression of LHPP, the coding sequence of LHPP was synthesized and inserted into the pCDH vectors. The packaging vectors PSPAX2 and PDM2G were co-transfected with Control (pCDH empty vector) or LHPP (pCDH vector inserted with LHPP CDS) into the 293T cells for virus production. After 72 or 96 h, the supernatants were harvested and filtered through 0.45 μm filters. Then they were used to infect the SW780 and BIU87 cells for two times (2 days per time) and the cells were used for further experiments. The green fluorescence intensity indicated the infection efficiency.

For LHPP knockdown, shRNA against LHPP was designed and inserted into the PLL3.7 lentivirus vectors. The sequence of LHPP shRNA is 5′-CTACATGAAGGCGCTTGAGTA-3′. The packaging vectors VSVG, REV and pMDL were co-transfected with shCtrl (PLL3.7 control vector) or shLHPP (PLL3.7 vector inserted with shLHPP) into the 293T cells. After culturing for 72 or 96 h, the virus supernatants were obtained and filtered through 0.45 μm filters. Then the T24 and 5637 cells were infected by shCtrl or shLHPP virus for two times (2 days per time) and then subjected for further experiments. The green fluorescence intensity indicated the infection efficiency. All of the cells were infected with indicated lentivirus

### Real time-quantitative PCR (RT-qPCR) analysis

The human specimens or indicated cells were lysed in Trizol reagent (Invitrogen) and subjected to total RNA extraction. The RNA was used for reverse transcribing with the High Capacity cDNA Reverse Transcription Kit (Life Technologies, Thermo Fisher Scientific, U.S.A.). Real-time PCR reaction was performed using Light-Cycler 480 SYBR Green 1 Master (Roche, U.S.A.) on a Bio-rad machine. The qPCR primer sequences were listed as follow: LHPP forward: 5′-CTGTGTGGTAATTGCAGACGC-3′, and reverse:5′-TAGTAACGCCCTTTTCCCAGT-3′; Cyclin B1 forward: 5′-AATAAGGCGAAGATCAACATGGC-3′, and reverse:5′-TTTGTTACCAATGTCCCCAAGAG-3′; p21 forward: 5′-TGTCCGTCAGAACCCATGC-3′, and reverse:5′-AAAGTCGAAGTTCCATCGCTC-3′; p27 forward: 5′-TAATTGGGGCTCCGGCTAACT-3′, and reverse:5′-TGCAGGTCGCTTCCTTATTCC-3′; Bcl-2 forward: 5′-ACGGTGGTGGAGGAGCTCTT-3′, and reverse:5′-CGGTTGACGCTCTCCACAC-3′; Cyclin D1 forward: 5′-CAATGACCCCGCACGATTTC-3′, and reverse: 5′-CATGGAGGGCGGATTGGAA-3′; GAPDH forward: 5′-CTGGGCTACACTGAGCACC-3′, and reverse: 5′-AAGTGGTCGTTGAGGGCAATG-3′; β-actin forward: 5′-CATGTACGTTGCTATCCAGGC-3′, and reverse: 5′-CTCCTTAATGTCACGCACGAT-3′. GAPDH or β-actin serves as internal control.

### Western blot analysis

Total protein was extracted from the human tissues or BC cells using lysis buffer (Beyotime) containing protease and phosphatase inhibitors (Roche). The concentration of the protein was measured by BCA protein assay kit (Beyotime). A total of 40 μg protein was mixed with 2×loading buffer and subjected to sodium dodecyl sulfate polyacrylamide gel electrophoresis (SDS/PAGE) and polyvinylidenefluoride membranes (PVDF; Millipore, U.S.A.) transfering. The PVDF membranes were then blocked with 5% skim milk at room temperature for 1 h and blotted with indicated primary antibodies at 4°C overnight. After incubating with secondary antibodies for 2 h at romm temperature, the chemiluminesence analysis was determined using enhanced chemiluminescence (ECL, Thermo Fisher Scientific).

### Cell viability and growth measurement

The viability and growth of shCtrl and shLHPP T24 and 5637 cells, Ctrl and LHPP overexpressed SW780 and BIU87 cells were analyzed by CCK (Cell Counting Kit) assay and colony formation assay. For CCK assay, 2000 cells per well were seeded in 96-well plates containing 200 μl culture medium. The cell viability of 0 h as shown in the figures was measured 6 h after seeding. Each well was added with 20 μl CCK solution and incubated at 37°C for 2.5 h. The spectrophotometric absorbance of each well was measured at 450 nm.

For colony formation assay, equal number of the cells were seeded and cultured for 8 or 14 days. The plates were then washed by PBS fixed with methanol for 1 h. After stained with crystal violet solution for 30 min, the plates were washed with clean water and subjected to photographing.

### Glucose and lactate measurement

A total of 2*10^5^ shCtrl or shLHPP T24 and 5637 cells, and Ctrl or LHPP over-expressed SW780 and BIU87 cells were seeded in triplicate in 12-well plates. After culturing for 1 day, the cell number was measured and the culture medium was harvested. Then the culture medium was subjected to glucose or lactate detection using an Olympus AU5400 machine. The glucose consumption and lactate production were normalized to cell number.

### Statistical analysis

The data were presented as means ± SD of three independent experiments. Student’s *t* test was applied for the univariate analysis. Difference was considered statistically significant when *P*<0.05.

## Results

### LHPP is down-regulated in BC tissues and cells

**Figure 1:**
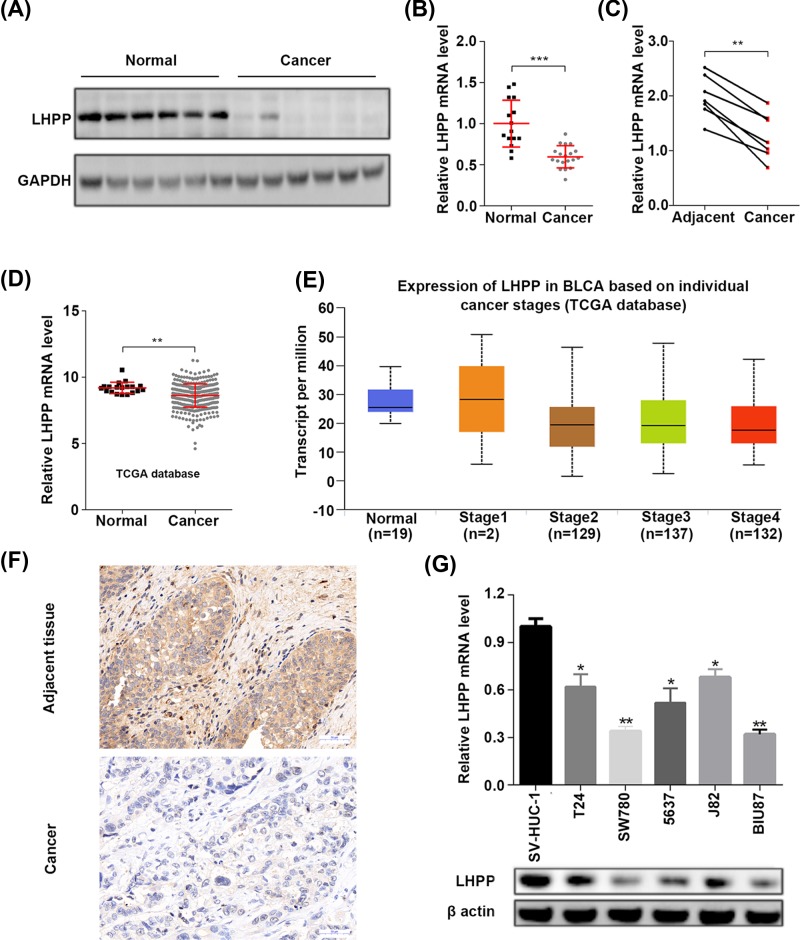
LHPP mRNA and protein abundance is reduced in BC tissues (**A**) BC and normal tissues (three of them were the adjacent normal tissues of the cancer samples) were subjected to Western blot analysis of LHPP. GAPDH serves as internal control. (**B**) Relative mRNA expression of LHPP in BC (*n*=19) and normal tissues (*n*=15, seven of them were the adjacent normal tissues of the cancer samples). ***P<0.001. (**C**) Relative mRNA expression of LHPP in BC and adjacent normal tissues (*n*=7). **P<0.01. (**D**) Relative mRNA level of LHPP in BC (*n*=407) and normal tissues (*n*=19). **P<0.01. (**E**) Relative mRNA level of LHPP in normal tissues (*n*=19), BC tissues of stage 1 (*n*=2), stage 2 (*n*=129), stage 3 (*n*=137) and stage 4 (*n*=132). **P<0.01 (normal vs stage 2, normal vs stage 3 and normal vs stage 4). (**F**) Immunohistochemistry analysis of LHPP in BC and adjacent normal tissues. Scale bar, 50 μm. (**G**) Relative mRNA and protein expression of LHPP in human normal uroepithelium cells SV-HUC-1 and BC cell lines T24, SW780, 5637, J82 and BIU87. *P<0.05, **P<0.01 (indicated BC cells vs SV-HUC-1 cells).

We firstly explored the clinical significance of LHPP in BC by checking the protein and mRNA abundance of LHPP. We showed that LHPP protein level was reduced in BC tissues as compared with normal tissues (Figure 1A). Consistently, the mRNA expression of LHPP was also down-regulated in BC tissues (Figure 1B). We further analyzed its expression in BC tissues and adjacent normal tissues. The results showed that LHPP mRNA level was decreased in BC tissues (Figure 1C). To verify our results, the expression of LHPP was analyzed from the TCGA database. Consistently, the mRNA level of LHPP was reduced in BC tissues comparing with normal tissues (Figure 1D). We also analyzed the association between LHPP expression and tumor stage in the TCGA data. The results showed that compared with the normal tissues, the tumor tissues of stage 2, 3 and 4 had lower expression of LHPP, whereas no difference was observed between various stages (Figure 1E). Furthermore, immunohistochemistry analysis showed that LHPP expression was reduced in BC tissues as compared with the adjacent tissues (Figure 1F). In addition, the expression of LHPP was lower in various BC cell lines, including T24, SW780, 5637, J82 and BIU87, than in normal uroepithelium cell line SV-HUC-1 (Figure 1G). Collectively, LHPP might be correlated with bladder tumorigenesis.

### Down-regulated LHPP contributes to BC cell proliferation and growth

**Figure 2:**
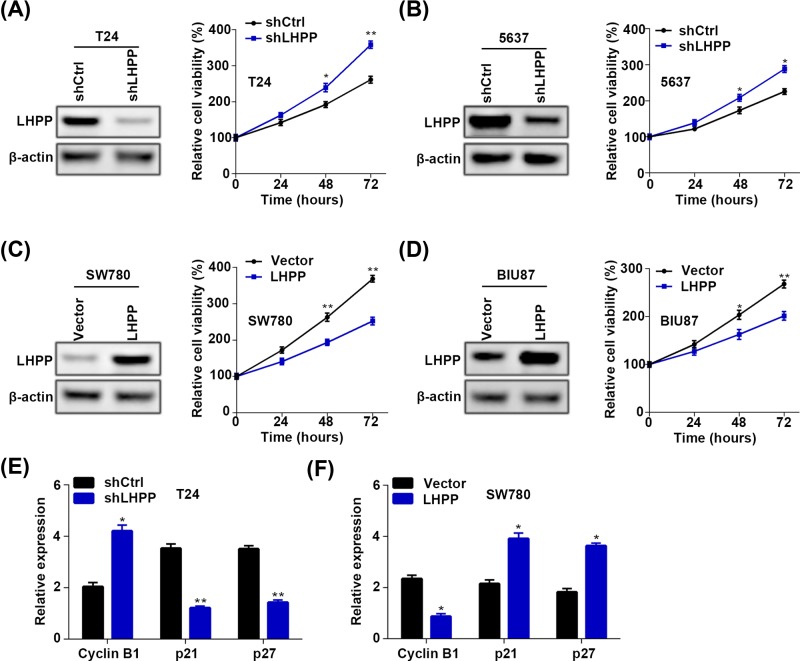
LHPP reduction promotes the proliferation of BC cells (**A**) shCtrl and shLHPP T24 cells were subjected to Western blot analysis of LHPP and CCK analysis of cell proliferation. *P<0.05 (shCtrl vs shLHPP at 48 h), **P<0.01 (shCtrl vs shLHPP at 72 h). (**B**) shCtrl and shLHPP 5637 cells were subjected to Western blot analysis of LHPP and CCK analysis of cell proliferation. *P<0.05 (shCtrl vs shLHPP at 48 and 72 h). (**C**) Empty vector (Vector) and LHPP overexpressed (LHPP) SW780 cells were subjected to Western blot analysis of LHPP and CCK analysis of cell proliferation. **P<0.01 (Vector vs LHPP at 48 and 72 h). (**D**) Empty vector (Vector) and LHPP overexpressed (LHPP) BIU87 cells were subjected to Western blot analysis of LHPP and CCK analysis of cell proliferation. *P<0.05 (Vector vs LHPP at 48 h), **P<0.01 (Vector vs LHPP at 72 h). (**E**) shCtrl and shLHPP T24 cells were subjected to qRT-PCR analysis of cyclin B1, p21 and p27. *P<0.05, **P<0.01 (shCtrl vs shLHPP). (**F**) Empty vector (Vector) and LHPP overexpressed (LHPP) SW780 cells were subjected to qRT-PCR analysis of cyclin B1, p21 and p27. *P<0.05, **P<0.01 (Vector vs LHPP).

**Figure 3:**
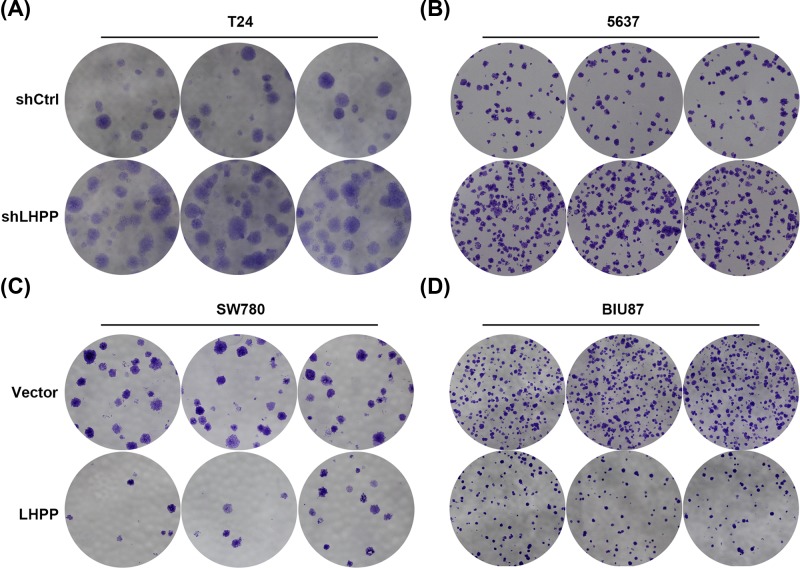
LHPP inhibits the colony formation of BC cells (**A,B**) shCtrl and shLHPP T24 (A) and 5637 (B) cells were subjected to colony formation assay. (C and D) Empty vector (Vector) and LHPP overexpressed (LHPP) SW780 (**C**) and BIU87 (**D**) cells were subjected to colony formation assay.

We next investigated the role of LHPP in BC cell proliferation using lentivirus-mediated knockdown and overexpression. Since the T24 and 5637 cells had relatively higher LHPP expression than the SW780 and BIU87 cells, we knocked down LHPP in T24 and 5637 cells and overexpressed LHPP in SW780 and BIU87 cells. Western blots showed that LHPP was efficiently down-regulated in T24 and 5637 cells. The viability of T24 and 5637 cells was enhanced by LHPP silencing as shown by the CCK assay (Figure 2A,B). By contrast, LHPP ectopic expression suppressed the proliferation of SW780 and BIU87 cells (Figure 2C,D). qRT-PCR assay showed that cyclin D1 was negatively, whereas p21 and p27 was positively regulated by LHPP in BC cells (Figure 2E,F). To confirm our results, we performed colony formation assay in these cells. Consistently, LHPP silencing resulted in accelerated colony formation of both T24 and 5637 cells, while LHPP overexpression reduced the colony numbers of SW780 and BIU87 cells (Figure 3). This indicates that LHPP suppresses the BC cell proliferation and growth at least partly through regulating cell-cycle proteins. Taken together, down-regulated LHPP promoted BC cell proliferation and growth.

### LHPP suppresses the glycolysis of BC cells

**Figure 4:**
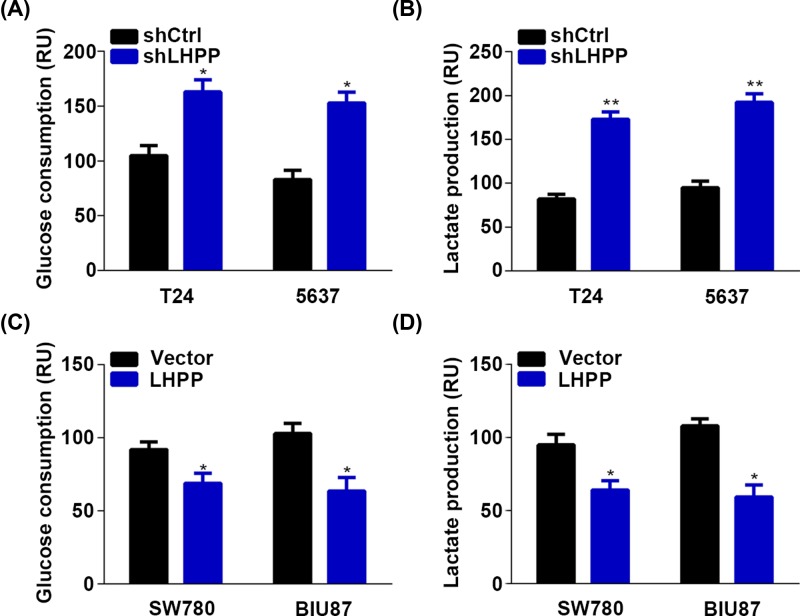
Glycolysis is suppressed by LHPP in BC cells (**A,B**) Glucose consumption (A) and lactate production (B) in shCtrl and shLHPP T24 and 5637 cells. *P<0.05, **P<0.01 (shCtrl vs shLHPP in T24 or 5637 cells). (C and D) Glucose consumption (**C**) and lactate production (**D**) in empty vector (Vector) and LHPP overexpressed SW780 and BIU87 cells. *P<0.05 (Vector vs LHPP in T24 or 5637 cells).

Aerobic glycolysis is a hallmark of cancer. Cancer cells prefer to metabolize glucose into lactate even when oxygen is sufficient [9]. Next, we determined whether LHPP regulates glycolysis in BC cells by measuring the glucose and lactate level. We showed that LHPP repressed the glycolysis as indicated by the increased glucose consumption and lactate production in LHPP silencing T24 and 5637 cells and decreased glucose consumption and lactate production in LHPP overexpressed SW780 and BIU87 cells (Figure 4). These results suggested that LHPP inhibited glycolysis in BC cells.

### LHPP reduction activates p65 signaling pathway depending on AKT activity

**Figure 5:**
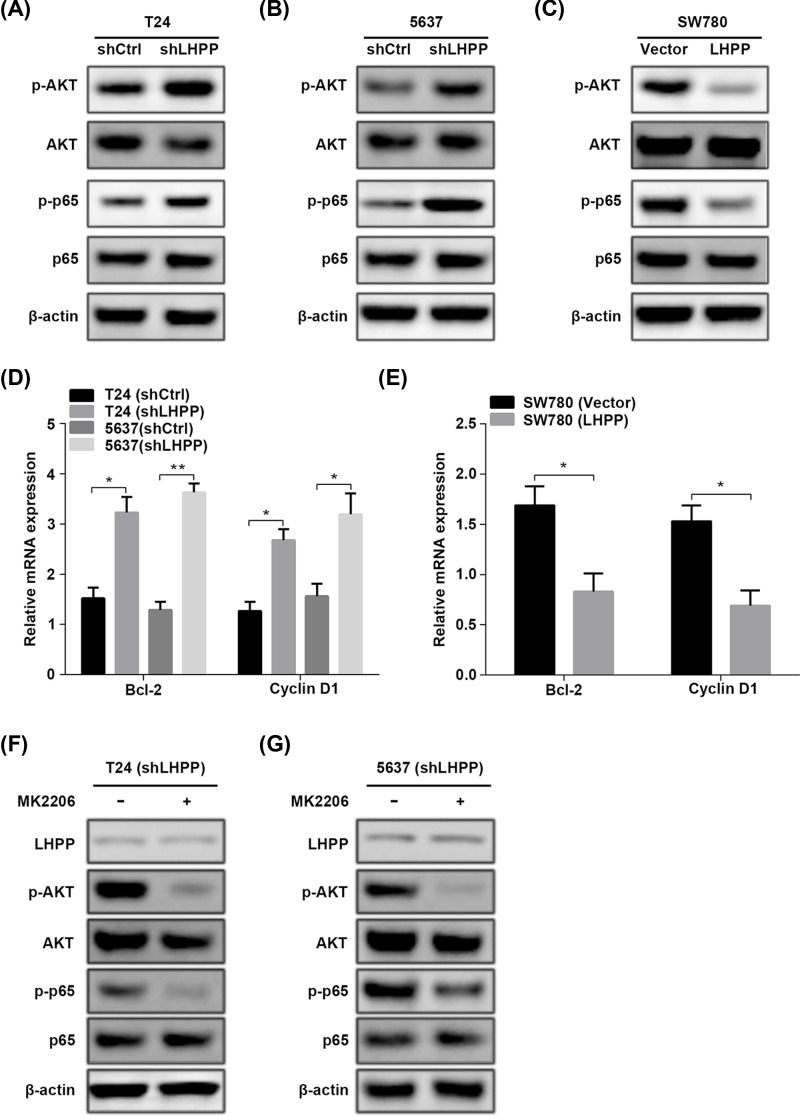
LHPP inactivates p65 signaling pathway through AKT activity (**A**–**C**) Western blot analysis of LHPP, p-AKT, AKT, p-p65 and p65 in shCtrl and shLHPP T24 cells (A) or 5637 cells (B) and in empty vector (Vector) and LHPP overexpressed SW780 cells (C). (**D**) qRT-PCR analysis of Bcl-2 and cyclin D1 in cells described in (A) and (B). (**E**) qRT-PCR analysis of Bcl-2 and cyclin D1 in cells described in (C). (**F** and **G**) shLHPP T24 (F) or 5637 (G) cells were treated with or without AKT inhibitor MK2206 and subjected to Western blot analysis of p-AKT (Ser473), AKT, p-p65 (Ser536) and p65.

It has been reported that LHPP silencing leads to activation of PI3K/AKT signaling pathway in both HCC and cervical cancer. We therefore checked whether AKT was regulated by LHPP in BC. As expected, LHPP silencing caused increased phosphorylation of AKT in both T24 and 5637 cells. We also observed that phosphorylated p65 was enhanced in LHPP knockdown T24 and 5637 cells (Figure 5A,B). By contrast, the phosphorylation of AKT and p65 was suppressed by LHPP overexpression in SW780 cells (Figure 5C). The downstream targets of p65, Bcl-2 and cyclin D1, were up-regulated in LHPP silenced T24 and 5637 cells. In contrast, they were down-regulated in LHPP over-expressed SW780 cells (Figure 5D,E). To identify whether or not locates upstream of p65, shLHPP T24 and 5637 cells were treated with AKT inhibitor MK2206. Western blot results showed that MK2206 had no effect on LHPP expression, while it suppressed the phosphorylated AKT and p65 (Figure 5F,G), suggesting that AKT regulates p65 in LHPP silencing BC cells. Our results reveal that down-regulation of LHPP activates p65 through potentiating AKT signaling.

### LHPP expression in BC cells confers different sensitivity to p65 inhibitor

**Figure 6:**
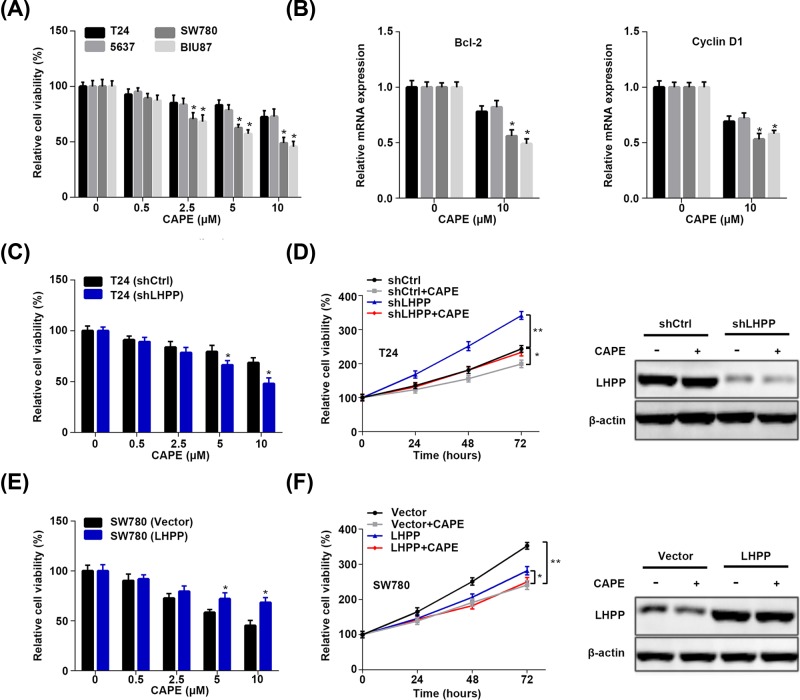
LHPP abundance in BC cells determines the inhibitory effect of p65 inhibitor CAPE (**A**) T24, 5637, SW780 and BIU87 cells were treated with different concentrations of p65 inhibitor CAPE for 72 h and the cell viability was determined by CCK assay. *P<0.05 (2.5 vs 0, 5 vs 0 and 10 vs 0 in SW780 and BIU87 cells). (**B**) T24, 5637, SW780 and BIU87 cells were treated with or without CAPE (10 μM) for 72 h and qRT-PCR was performed to analyze the Bcl-2 and cyclin D1 expression. *P<0.05 (10 vs 0 in SW780 and BIU87 cells). (**C**) shCtrl and shLHPP T24 cells were incubated with various concentrations of CAPE for 72 h and the cell viability was measured by CCK assay. *P<0.05 (shCtrl vs shLHPP). (**D**) shCtrl and shLHPP T24 cells incubated with or without (10 μM) for indicated time were subjected to CCK analysis of cell viability. The cells at 48 h were subjected to Western blot analysis of LHPP. *P<0.05 (shCtrl vs shCtrl+CAPE), **P<0.01 (shLHPP vs shLHPP+CAPE). (**E**) Empty vector (Vector) and LHPP overexpressed SW780 cells were incubated with various concentrations of CAPE for 72 h and the cell viability was analyzed by CCK assay. *P<0.05 (Vector vs LHPP). (**F**) Empty vector (Vector) and LHPP overexpressed SW780 cells treated with or without (10 μM) for indicated time were subjected to CCK analysis of cell viability. The cells at 48 h were subjected to Western blot analysis of LHPP. *P<0.05 (LHPP vs LHPP+CAPE), **P<0.01 (Vector vs Vector+CAPE).

To investigate whether LHPP regulation of p65 participates in BC cell proliferation, we used p65 inhibitor CAPE to treat BC cell and analyzed the cell proliferation and the downstream targets of p65. We observed that SW780 and BIU87 cells, which had lower expression LHPP, exhibited higher sensitivity to CAPE treatment (2.5, 5 and 10 μM) than LHPP highly expressed T24 and 5637 cell (Figure 6A). The down-regulation of p65 downstream targets, Bcl-2 and cyclin D1, was more obvious in SW780 and BIU87 cells than in T24 and 5637 cells (Figure 6B). Then we treated shCtrl or shLHPP T24 cells with various concentrations of CAPE. Interestingly, shLHPP T24 cells were more sensitive to CAPE treatment (Figure 6C). Likewise, CAPE treatment at different time more prominently suppressed the viability of shLHPP T24 cells than that of shCtrl cells (Figure 6D). Conversely, SW780 cells with LHPP ectopic expression were more resistant to CAPE treatment at the dosage of 5 and 10 μM (Figure 6E). At various time, the inhibitory effect of CAPE on SE780 cell proliferation was relatively lower in LHPP overexpressed cells (Figure 6F). Furthermore, CAPE treatment did not change the expression of LHPP in these cells (Figure 6D,F). Our results indicate that LHPP expression dictates the sensitivity of BC cells to p65 treatment.

## Discussion

Phosphatase, which dephosphorylates the kinases and other downstream substrates, plays a key role in controlling signaling transduction and cell fate. Dys-regulation of protein-tyrosine phosphatases is commonly observed in a large amount of cancers [10,11]. The well-known protein-tyrosine phosphatase is Phosphatase and tensin homolog (PTEN) that represents as the second most frequently altered tumor suppressor in cancer, after p53 [12]. On the contrary, Phosphatase of Regenerative Liver (PRL) family members are highly expressed and serve as oncogenes in various cancers [10]. However, beyond the protein-tyrosine phosphatases, there is still lack of evidence addressing the role of other kinds of phosphatases in cancer development. In the present study, we revealed the tumor suppressive role of LHPP in BC.

LHPP gene locates at the chromosome 10. It is ubiquitously expressed in brain, kidney, liver and urinary bladder tissues [13]. A genome-wide association study has revealed a single-nucleotide polymorphism (SNP) at the LHPP gene (rs35936514) correlated with major depressive disorder [6]. Recently, the involvement of LHPP in cancer development has been discovered. Genome-wide association study has identified 10q26.13 (rs201982221, LHPP) as the significantly associated loci in the oral and pharyngeal cancers [14]. Hindupur et al. [5] showed that LHPP abundance was decreased in HCC specimens and its low expression predicted poor disease-free survival and overall survival. In mouse model, LHPP ectopic expression reversed the liver tumorigenesis of hyper-active AKT and mTOR, suggesting that AKT/mTOR signaling pathway promotes HCC by repressing LHPP. LHPP is also a tumor suppressor in cervical cancer. It is down-regulated in the majority of cervical cancer tissues and its low expression is correlated with the short survival of the patients. LHPP knockdown accelerates the proliferation and metastasis of cervical cancer [8]. Here, the protein and mRNA level of LHPP were reduced in BC tissues. TCGA database also showed that LHPP was down-regulated in BC tissues. In addition, BC cells exhibited lower mRNA level as compared with the normal cells. Functional study demonstrated that LHPP reduction promoted the proliferation and colony formation of BC cells. In contrast, LHPP overexpression had an inverse effect on BC cell viability. Aerobic glycolysis, which represents Warburg’s effect, is a metabolic hallmark of cancer [9,15]. Even in the presence of oxygen, cancer cells prefer to metabolize the glucose in the glycolytic way. Here, we showed that glycolysis was potentiated by LHPP knockdown. Glucose consumption and lactate production were enhanced in LHPP silencing BC cells, while they were reduced in LHPP overexpressed BC cells. This phenomenon suggested that LHPP silencing reinforced the cancer characteristic of BC. Furthermore, the cell cycle factors, such as cyclin B, p21 and p27 were also mediated by LHPP. Our study reveals that LHPP is a potential tumor suppressor in BC.

PTEN/PI3K/AKT signaling pathway directly or indirectly participates in different cancer development [16]. Dys-regulation of any components of this pathway causes uncontrolled cell proliferation, suppressed apoptosis, altered cell metabolism and tumorigenesis [17–19]. Many substrates are identified as downstream targets of this pathway, such as p27, FOXO and caspase 9 [20]. Furthermore, this pathway also contributes to the tumor suppressive or oncogenic functions of other factors in cancers. For instance, LncRNA LINC00641 suppresses BC development via miR-197-3p/KLF10/PTEN/PI3K/AKT cascade [21]. miR-410-3p triggers prostate cancer progression through regulation of PTEN/AKT/mTOR signaling pathway [22]. Previous studies have shown that LHPP knockdown and overexpression results in increased and decreased phosphorylation of AKT in cervical cancer, respectively [8]. However, how LHPP negatively regulates AKT phosphorylation is not investigated. Moreover, AKT activation enhances p65 activity in various cancers [23–25]. In the present study, LHPP silencing led to activation of AKT signaling. The activity of p65, as well as the expression of p65 downstream targets, Bcl-2 and Cyclin D1 [26,27], was enhanced by LHPP knockdown. Importantly, AKT inhibition by MK2206 repressed the phosphorylation of p65. Our results suggest that LHPP negatively regulates p65 through AKT. NF-κB (p65) is a two-edged sword in cancer development. Although most studies show that activation of p65 promotes tumor development, inactivation of NF-κB accelerates chemical-induced HCC [28,29]. Therefore, whether p65 functions as an oncogene in BC with LHPP reduction should be addressed. Using p65 inhibitor CAPE, we found that p65 blockage exhibited different inhibition on the viability of BC cells that relatively higher sensitivity was observed in LHPP lowly expressed BC cells. In addition, LHPP silencing increased the sensitivity of BC cells to CAPE treatment at various concentrations and times, while inverse results were found in LHPP overexpressed BC cells. We also demonstrated that MK2206 and CAPE had no effect on LHPP expression. This indicated that the inhibitory effect of MK2206 and CAPE on BC cell proliferation was not through up-regulating LHPP. Since targeted therapies against tumor suppressor are difficult to develop, these findings may provide an effective strategy for BC or other cancer treatment which harbors loss of LHPP.

In conclusion, we provide for the first time that LHPP was down-regulation in BC tissues and its reduction promoted BC cell proliferation and growth through AKT/p65 signaling cascade. Therefore, LHPP expression can be a valuable biomarker for BC. Targeting p65 may benefit for BC patients with lowly expressed LHPP.

## Abbreviations

BC: bladder cancer
CAPE: caffeic acid phenethyl ester
CCK: Cell Counting Kit
HCC: hepatocellular carcinoma
LHPP: Phospholysine phosphohistidine inorganic pyrophosphate phosphatase
PRL: Phosphatase of Regenerative Liver
PTEN: Phosphatase and tensin homolog
PVDF: polyvinylidenefluoride membranes
RT-qPCR: Real time-quantitative PCR
SNP: single-nucleotide polymorphism
